# *Legionella pneumophila* Subspecies *fraseri* Infection after Allogeneic Hematopoietic Stem Cell Transplant, China

**DOI:** 10.3201/eid2804.211433

**Published:** 2022-04

**Authors:** Xiaojuan Wang, Yifan Guo, Yawei Zhang, Qi Wang, Shuo Yang, Hua Yang, Tianyi Wang, Hui Wang

**Affiliations:** Peking University People’s Hospital, Beijing, China (X. Wang, Y. Guo, Y. Zhang, Q. Wang, S. Yang, H. Wang);; Peking University Health Science Center, Beijing (Y. Guo, T. Wang);; Zhongshan Torch Development Zone Hospital, Zhongshan, China (H. Yang)

**Keywords:** Legionnaires’ disease, *Legionella pneumophila* subsp. *fraseri*, bacteremia, pneumonia, allogeneic hematopoietic stem cell transplant, aHSCT, bacteria, respiratory infections, China

## Abstract

We describe an immunosuppressed patient with bacteremia and pneumonia caused by *Legionella pneumophila* subspecies *fraseri* in China. We confirmed this diagnosis by using nanopore sequencing of positive blood cultures and subsequent recovery from buffered-charcoal yeast extract culture. Nanopore sequencing is an effective tool for early diagnosis of atypical infections.

*Legionella pneumophila* is an opportunistic atypical pathogen of community-acquired or hospital-acquired pneumonia (*1*–*3*). Underestimates of its prevalence are likely because the *Legionella* urinary antigen testing and buffered-charcoal yeast extract (BCYE) culture routinely used for diagnosis are sometimes available only in a few tertiary hospitals that specialize in respiratory diseases. Therefore, early diagnosis and prompt therapy for *L. pneumophila* infection are crucial. We report a case of *L. pneumophila* subspecies *fraseri* bloodstream infection in a patient in China after allogeneic hematopoietic stem cell transplant (aHSCT). We confirmed this diagnosis by using nanopore sequencing of positive blood cultures and by recovering *L. pneumofila* using BCYE medium. Chest radiography and computed tomography (CT) suggested acute *L. pneumophila* pneumonia. The study was approved by the Institutional Review Board of the Peking University People’s Hospital (approval no. 2019PHB134–01).

A 55-year-old man with acute T/myeloid mixed-cell leukemia was hospitalized because of nasal bleeding on day 62 after aHSCT. After treatment for thrombocytopenia, he experienced mild diarrhea on day 101. Three days later (day 104), his temperature was 38.8°C (Appendix Figure 1), but he had no accompanying symptoms. One aerobic blood culture vial, 1 anaerobic blood culture vial, and 1 Myco/F blood culture vial (BD, https://www.bd.com) per puncture point (2 puncture points) were collected for culture. Chest radiography and CT displayed multiple solid nodules in both lungs; the largest was in the right lower lobe, which suggested infection (Figure). The patient then experienced a 3-day continuous high fever with a maximum temperature of 39.5°C, a small amount of white sputum, and a blood oxygen saturation of 90% on day 107; the same number of blood culture vials were collected. CT showed a large, solid, fuzzy shadow with a unclear boundary, uneven internal density, and low-density plaques in the lower lobe of the right lung (Figure). The patient was administered linezolid (0.6 g every 12 h) and imipenem (0.5 g every 8 h) to control the infection. The patient’s temperature decreased to a normal level, but the C-reactive protein values did not decrease to reference range (Appendix Figure 2).

**Figure:**
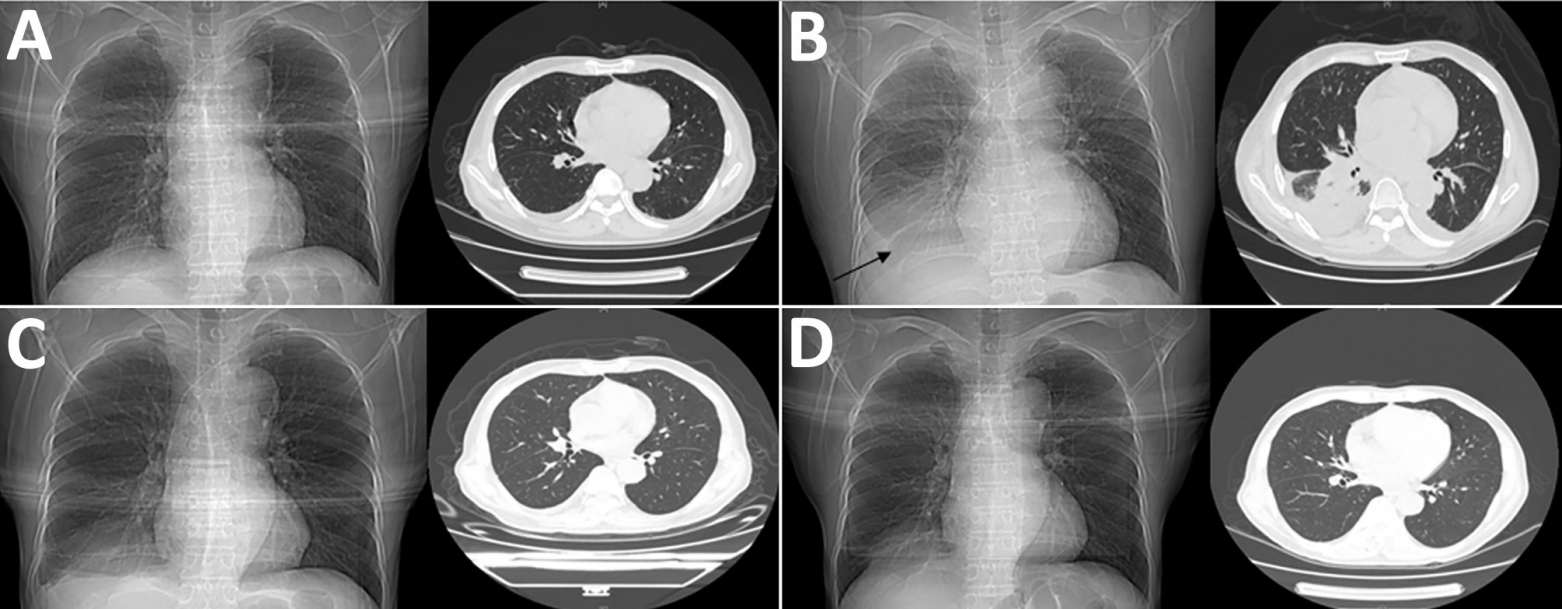
Chest radiography and computed tomography images during acute infection with *Legionella pneumophila* subspecies *fraseri* after allogeneic hematopoietic stem cell transplant, China, 2021. A) May 13 (day 104), B) May 17 (day 108), C) May 24 (day 115), D) June 1 (day 123). Arrow in panel B indicates a large, solid, fuzzy shadow with unclear boundary, uneven internal density, and low-density plaques in the lower lobe of the right lung.

After ≈10 days (256 h) of incubation, we observed that the blood cultures in the Myco/F vial collected on day 104 after aHSCT, which are commonly used to culture *Mycobacterium* spp. and fungus, contained the only growing gram-negative bacilli. We then transferred the positive blood cultures to blood nutrition plates for further culture in 5% carbon dioxide, anaerobic, and microaerobic environments, but this culture failed. Simultaneously, we directly used a serum separator-gel tube and matrix-assisted laser desorption/ionization time-of-flight (MALDI-TOF) mass spectrometry (*4*) to identify positive blood cultures but were unsuccessful. Subsequently, we extracted DNA from the positive blood cultures for nanopore sequencing (*5*,*6*); *L. pneumophila* subsp. *fraseri* was detected after 1 hour (Appendix; BioProject Short Read Archive accession no. PRJNA744850). The coverage length and depth were 37.67% and 2.04 with 1,337 raw reads. After timely communication of the sequencing results to the clinical department, the patient was administered azithromycin (0.5 g 1×/d for 1 d) , followed by moxifloxacin (0.4 g 1×/d for 9 d) to ease symptoms of discomfort caused by azithromycin. The infection was finally controlled, and diarrhea symptoms improved 3 days after appropriate moxifloxacin therapy was initiated.

Afterward, we analyzed serum collected on May 24 (day 114) and May 27 (day 117) and detected *L. pneumophila* IgM by using an ELISA kit (Euroimmun, https://www.euroimmun.com). *Legionella* urinary antigen testing was not performed. Meanwhile, 100 μL of the positive blood cultures was inoculated on the in-house BCYE agar supplied with *Legionella* BCYE growth supplement medium (OXOID; Thermo Fisher Scientific, https://www.thermofisher.com). Two days later, we successfully isolated *L. pneumophila* and displayed wet blue-purple luster colonies (Appendix Figure 3); *L. pneumophila* was finally identified using MALDI-TOF mass spectrometry. During the course of infection, no more sputum specimens were available because the patient had no obvious cough or expectoration.

The fatality rate of Legionnaires’ disease is between 5% and 30% (*7*). Risk factors for *L. pneumophila* infection include age >50 years, solid tumors or hematologic malignancies, solid organ transplant, and immunosuppression (*8*,*9*). Hence, shortening the turnaround time to identify microorganisms is crucial for timely diagnosis and appropriate therapy, which influence death rates. In this case, the diagnosis of *L. pneumophila* pneumonia was not possible on the basis of the atypical radiologic evidence, high fever (>38°C), elevated C-reactive protein, and diarrhea, although such manifestations are the most common symptoms of *L. pneumophila* infections (*10*). Furthermore, administration of corticosteroids and immunosuppressive drugs likely obscured the respiratory symptoms. Rapid nanopore sequencing with a short turnaround time has the potential to effectively expedite the detection of *L. pneumophila* infection, guaranteeing the appropriate antibiotic therapy, especially for immunosuppressed patients with atypical symptoms.

Appendix Additional information about *Legionella pneumophila* subspecies *fraseri* infection after allogeneic hematopoietic stem cell transplant, China
