# Supplementary material for: Legionella pneumophila Subspecies fraseri Infection after Allogeneic Hematopoietic Stem Cell Transplant, China
Source: Emerg Infect Dis. 2022 Apr;28(4):903–5. doi: 10.3201/eid2804.211433 (PMC8962907; doi:10.3201/eid2804.211433)
Supplement: Appendix — Additional information about Legionella pneumophila subspecies fraseri infection after allogeneic hematopoietic stem cell transplant, China [file 21-1433-Techapp-s1.pdf]

Article DOI: https://doi.org/10.3201/eid2804.211433

Legionella pneumophila Subspecies *fraseri* Infection after Allogeneic Hematopoietic Stem Cell Transplant, China

Appendix

Nanopore Sequencing and Bioinformatic Analysis Pipeline

A nanopore sequencing method that could detect unknown pathogens in 1 hour was adopted to identify the unknown Gram-negative bacilli from the positive blood cultures. The total DNA extraction and libraries preparation steps take 5–6 hours. The total DNA was extracted from 3 mL of positive blood cultures by using the Quick-DNA/RNA TM Viral Kit (Zymo Research, https://www.zymoresearch.com). Sterile deionized water was used as the external negative control. The DNA concentration was determined with the Qubit dsDNA High-Sensitivity (HS) Assay Kit (ThermoFisher Scientific, https://www.thermofisher.com). The ONT libraries were prepared using the PCR Barcoding Kit (SQK-PBK004, Oxford Nanopore Technologies, https://nanoporetech.com). Sequencing was performed using an Oxford Nanopore GridlON X5 instrument (Oxford Nanopore Technologies). The real-time sequencing process was controlled using ONT MinKNOW software version 3.6.5 (Oxford Nanopore Technologies).

The ONT Guppy software version 3.2.10 was applied to process raw sequenced data (fast5 files) into reads (fastq files). Then, the sequencing reads were demultiplexed using the blastn-short (version 2.7.1+) and the lastal (version 980) software, followed by removal of the short (read length ≤500 nt) and low-quality reads (mean q-score ≤8). Subsequently, the host reads were eliminated by aligning them to the human reference genome (GRCh38) using the minimap2 software (version 2.14-r883). The remaining reads were assigned to taxonomy using

Centrifuge software (version 1.0.4) and validated using megablast software (version 2.10.0+). Reads with alignments to multiple bacterial species were excluded from further analyses. Abundance was calculated as the number of reads of a microbe divided by the total number of reads of all microbes.

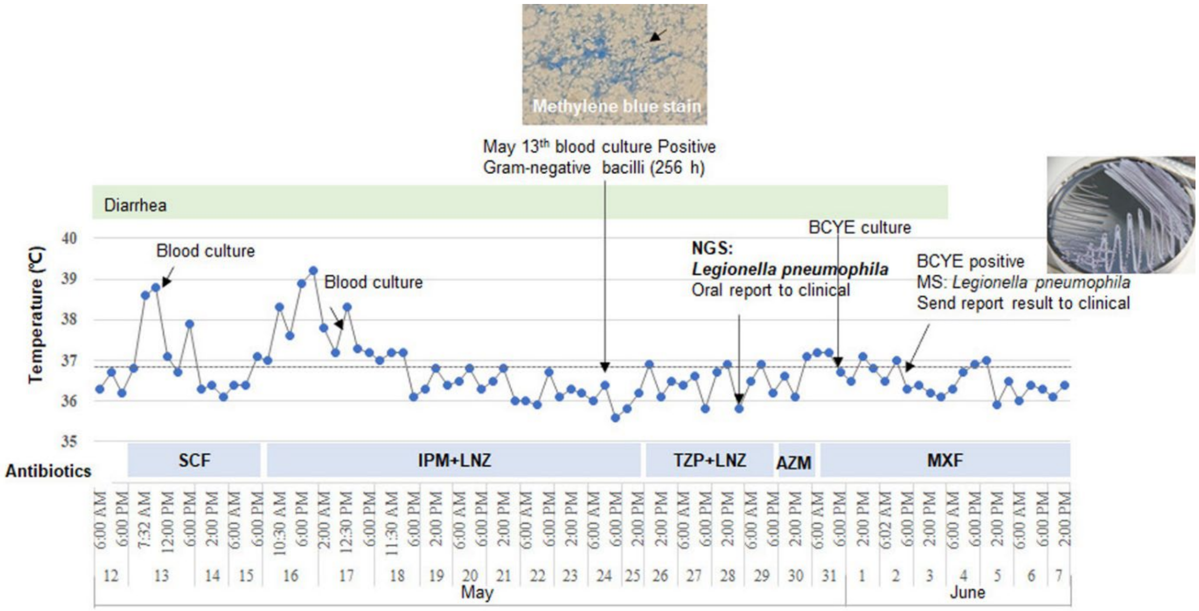

Appendix Figure 1. Temperature, symptoms, and antibiotic therapy of patient with acute infection with *Legionella pneumophila* subsp. *fraseri* after allogeneic hematopoietic stem cell transplantation, China, May 12, 2021–June 7, 2021. The microscopic morphology with methylene blue staining of the positive blood cultures was based on 1 drop (1 mL syringe) syringed from positive blood cultures followed by methylene blue staining. AZM, azithromycin; BCYE, buffered-charcoal yeast extract; IPM, imipenem; LNZ, linezolid; MXF, moxifloxacin; MS, matrix-assisted laser desorption ionization time-of-flight mass spectrometry; SCF, cefoperazone/sulbactam; TZP, piperacillin/tazobactam.

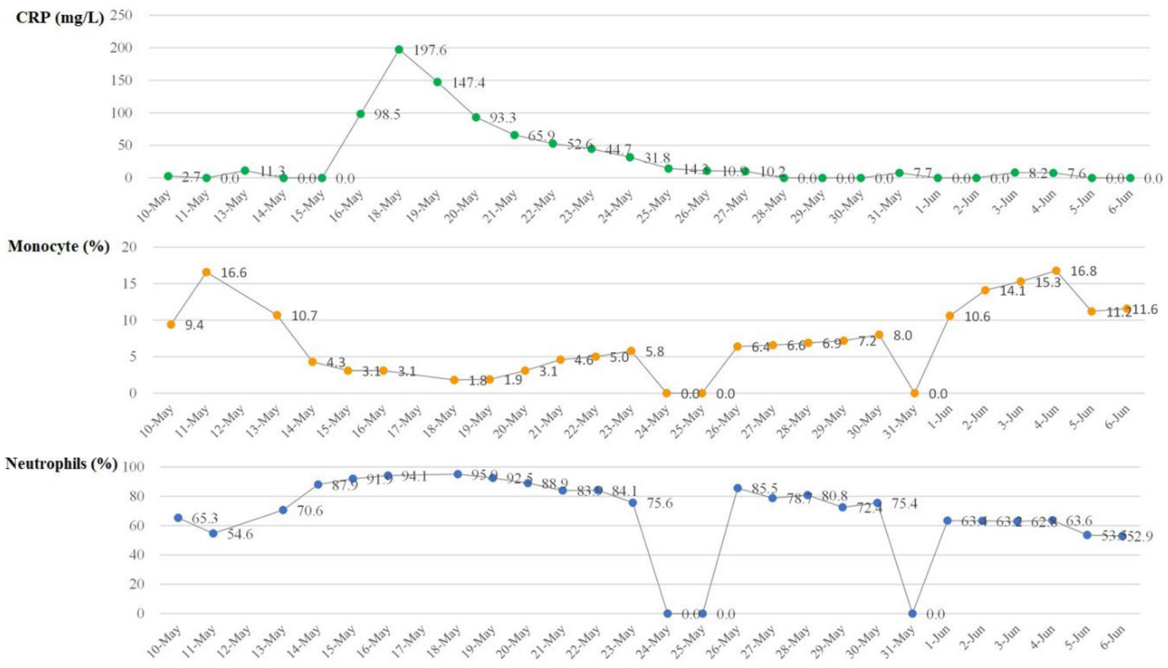

	10-May	11-May	13-May	14-May	15-May	16-May	18-May	19-May	20-May	21-May	22-May	23-May	24-May	25-May	26-May	27-May	28-May	29-May	30-May	31-May	1-Jun	2-Jun	3-Jun	4-Jun	5-Jun	6-Jun
CRP (mg/L)	2.7		11.3			98.5	197.6	147.4	93.3	65.9	52.6	44.7	31.8	14.3	10.9	10.2				7.7			8.2	7.6		
Monocyte (%)	9.4	16.6	10.7	4.3	3.1	3.1	1.8	1.9	3.1	4.6	5.0	5.8			6.4	6.6	6.9	7.2	8.0		10.6	14.1	15.3	16.8	11.2	11.6
Neutrophils (%)	65.3	54.6	70.6	87.9	91.9	94.1	95.0	92.5	88.9	83.9	84.1	75.6			85.5	78.7	80.8	72.4	75.4		63.4	63.2	62.8	63.6	53.5	52.9

Appendix Figure 2. The C-reactive protein (CRP) levels, monocyte percentage and neutrophils percentage trends of patient during acute infection with *Legionella pneumophila* subsp. *fraseri* after allogeneic hematopoietic stem cell transplantation, China, May 10, 2021–June 6, 2021. Values marked with red boxes were higher than normal values. The normal value ranges of CRP, monocyte percentages, and neutrophils percentages are <8 mg/L, 3%–10% and 40%–75%.

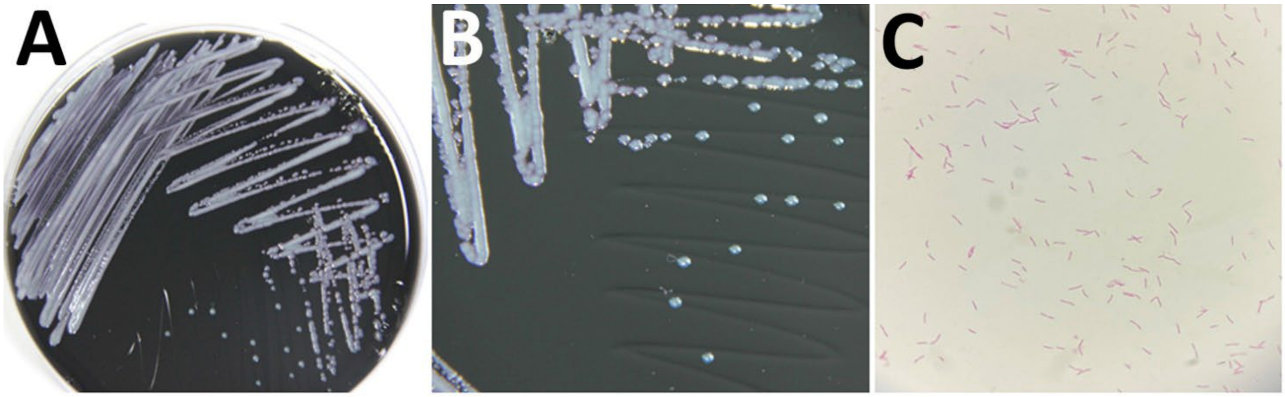
A
B
C
